# Comparative transcriptome analysis of PBMCs in cats diagnosed with and recovered from FIPV

**DOI:** 10.1186/s42826-025-00247-5

**Published:** 2025-06-13

**Authors:** Ju Young Lee, Hyeong Ryeol Cho, Hong-Geun Oh, Jeong Ho Hwang

**Affiliations:** 1 Center for Large Animals Convergence Research, Korea Institue of Toxicology, 30 Baekhak1- gil, Jeongeup, Jellabuk-do 56212 Republic of Korea; 2 Center for Companion Animal New Drug Development, Korea Institue of Toxicology, 30 Baekhak1-gil, Jeongeup, Jellabuk-do 56212 Republic of Korea; 3 Center for Animal Medicine and Foods, Korea Institue of Toxicology, 30 Baekhak1-gil, Jeongeup, Jellabuk-do 56212 Republic of Korea; 4 R&D Division, HUVET Co., Ltd, Hanaro, Iksan-si, Jellabuk-do 483-26 Republic of Korea

**Keywords:** Feline infectious peritonitis, GS-441524, mRNA sequencing

## Abstract

**Background:**

Feline infectious peritonitis is a viral disease caused by feline coronavirus an enveloped virus with a single-stranded RNA genome that is approximately 30 kb long. Although FCoV generally causes mild symptoms, approximately 5% of cases progress to death in cats worldwide. FCoV shares certain virological features with severe acute respiratory syndrome coronavirus 2 that causes COVID-19, indicating that common therapeutic strategies may be applicable. GS-441524 the parent drug of remdesivir and a competitive inhibitor of nucleoside triphosphates in viral RNA synthesis is a well-known treatment for FIP. However, comparative transcriptome and gene ontology analyses of normal (Normal), FIP-diseased (FIPD), and FIP-recovered (FIPR) cats have not yet been conducted.

**Results:**

In this study, we compared the mRNA expression profiles of peripheral blood mononuclear cells from Normal, FIPD, and FIPR cats to identify immunological alterations. We identified 677 (FIPD/Normal) and 431 (FIPR/FIPD) differentially expressed genes with statistical significance. These data were input into the bioinformatics program. As a result, the analysis revealed statistically significant and contrasting patterns of canonical pathways of neutrophil degranulation and interleukin-8 (IL-8) signaling pathways. Additionally, we observed that kruppel-like factor 6 (KLF6) and nuclear factor kappa-light-chain-enhancer of activated B cells (NF-κB) were upstream molecules of IL-8, promoting neutrophil activation and function.

**Conclusions:**

This study identified immunological alterations in PBMCs of Normal, FIPD, and FIPR cats. KLF-6 and NF-κB were found to regulate IL-8-mediated neutrophil activation.

**Supplementary Information:**

The online version contains supplementary material available at 10.1186/s42826-025-00247-5.

## Background

Feline coronavirus (FCoV) is a single-stranded RNA (ss-RNA) virus from the Coronaviridae family that is commonly present in cats worldwide [1]. Feline infectious peritonitis (FIP) is a viral disease caused by the feline infectious peritonitis virus (FIPV), which originates from a mutated form of the non-pathogenic subtype of FCoV, feline enteric coronavirus (FECV). FECV infects intestinal epithelial cells, causing mild symptoms, such as diarrhea, vomiting, and upper respiratory symptoms [2]. By contrast, FIPV targets monocytes and macrophages. These cells trigger the release of pro-inflammatory cytokines, such as tumor necrosis factor-alpha (TNF-α), interleukin-1 beta (IL-1β), adhesion molecules, and vascular endothelial growth factor, resulting in systemic inflammation [3].

GS-441524, an adenosine nucleoside analog, is considered a treatment for FIP, effectively treating FIP and significantly enhancing survival rates in FIP-infected cats [4, 5]. Intracellularly, it undergoes phosphorylation to form monophosphate, which is subsequently phosphorylated to its active triphosphate form, GS-443902 [6]. This active form inhibits viral replication by mimicking endogenous adenine and undergoing integration into the viral RNA [7]. However, the immune-related molecular and biological pathways altered during FIP progression and recovery induced by GS-441524 remain poorly understood.

To overcome these limitations, we performed transcriptomic analysis of PBMCs derived from Normal, FIPD, and FIPR cats, along with FIPD/Normal and FIPR/FIPD comparative analyses. These comparisons were further analyzed using bioinformatics tools, such as ingenuity pathway analysis (IPA) and Kyoto Encyclopedia of Genes and Genomes (KEGG) analysis to identify biological pathways involved in FIP progression and recovery. These results were confirmed at the mRNA level by reverse transcription quantitative PCR (RT-qPCR).

## Methods

### Animals

Three cats from each group were included in this study. Veterinarians diagnosed the FIP-infected cats and treated them with GS-441,524 for recovery. During the hospital visit, blood samples were collected from the cephalic vein, following the experimental protocol. All procedures involving animal subjects were approved by the Huvet Institutional Animal Care and Use Committee (IACUC; approval number; HV2022-004). All efforts were taken to reduce the cats used and to alleviate any potential pain or distress during the experiments.

### FIP diagnostic methods

The diagnosis of FIP was determined based on the following diagnostic criteria [8]: (1) evaluation of anorexia, (2) evaluation of pyrexia, (3) confirmation of exudative ascites through radiographic examination, (4) detection of a positive reaction in the Rivalta’s test using ascitic fluid sample, and (5) measurement of the Albumin/Globulin (A/G) ratio (< 0.4 considered as positive) in serum. These clinical and experimental criteria were integrated to diagnose effusive FIP.

### RNA isolation

PBMCs were isolated from 1 mL samples of whole blood collected in ethylenediaminetetraacetic acid tubes, based on the density gradient separation method with Ficoll (Histopaque reagent-1077; Sigma-Aldrich, USA). RNA (1 µg) was extracted from 3 × 10^6^ PBMCs obtained from normal and obese cats using the phenol/chloroform extraction method, with the integrity of the isolated RNA being assessed using an Agilent 2100 Bioanalyzer (Agilent, CA, USA).

### Library Preparation and sequencing

For control and test RNAs, we constructed the respective libraries using a QuantSeq 3’- mRNA-Seq Library Prep Kit FWD (Lexogen) according to the manufacturer’s instructions. Briefly, the respective total RNAs were prepared and oligo-dT primers containing an Illumina-compatible sequence at the 5’- ends were hybridized to the RNA, which was subsequently reverse transcribed. Following degradation of the RNA template, second-strand synthesis was initiated using a random primer containing an Illumina-compatible linker sequence at the 5’- end. The double-stranded libraries were purified using magnetic beads to remove all reaction components, and were subsequently amplified to attach the complete adapter sequences required for cluster generation, and thereafter were purified to remove the PCR components. High-throughput sequencing was performed as single-end 75-bp sequencing using a NextSeq 500 / 550 system (Illumina Inc.)

### Data processing

Reads were filtered using BBDuk to quality-trim both ends to Q20 and aligned to the Felis_catus_9.0 genome assembly sequence using Bowtie 2 [9]. The alignment file was used for assembling transcripts and estimating their abundance. Differentially expressed genes (DEGs) were determined based on counts from unique and multiple alignments using coverage in Bedtools [10]. Analysis of DEGs between groups, along with the generation of hierarchical heat maps and volcano plots, was performed using Excel-based differentially expressed gene analysis software (ExDEGA; ebiogen, Inc., Seoul, Korea). The read count data were processed based on the TMM + CPM normalization method using EdgeR within R [11] using Bioconductor [12].

### Functional annotation and gene ontology analyses

To assess the function of DEGs among the FIPD, Normal, and FIPR groups, we performed KEGG pathway analysis using the Database for Annotation, Visualization, and Integrated Discovery (DAVID) Bioinformatics Resources 6.8 [13, 14]. Additionally, upstream regulators, including transcription factors, canonical pathways, and causal networks were analyzed using IPA (Qiagen, CA, USA).

### RT-qPCR

RT-qPCR was conducted in a 20 µl reaction mixture including SYBR Green Chemistry (Bio-Rad, CA, USA), 0.5 µM of each primer, and template cDNA. Amplifications were carried out using the Quantstudio 5 Real-time PCR System (Applied Biosystems, CA, USA). The amplification conditions consisted of an initial denaturation step at 95°C for 3 min, followed by 40 cycles of denaturation at 95°C for 20 s, and annealing and elongation at 60°C for 1 min. A final elongation step was not included. Glyceraldehyde 3-phosphate dehydrogenase (GAPDH) was used as the endogenous control to normalize the RNA samples. Primer sequences are detailed in Table 1. Relative gene expression levels were calculated using the 2^−ΔΔCt^ method and presented as log_2_ fold change (log_2_ FC).

**Table 1:** Primer used for quantitative real-time PCR

Gene symbol	Prime sequences (from 5’ to 3’)	Length (bp)	Accession no.
*IL-8*	F: GACCCCAAGCAAAAGTGGGT	172	NM_001009281.1
	R: ACTGCATGAAGTGCTGAAGTG		
*TLR8*	F: CGCCATTTCCCTGTACACCT	74	XM_045050678.1
	R: CGTCAGCGTTTGGAAAGCAA		
*TLR4*	F: GCTGGCAATTCTTTCCAGGACAAC	208	NM_001009223.1
	R: TCTGGAGGGAGTGAAGAGGTTCAT		
*MyD88*	F: TGTCCACGTGCTGGGTTATC	117	XM_003992253.5
	R: GGAGAGACCGAGGCCTAAGA		
*KLF6*	F: GTCTGGCTGGCCTGGTATTT	196	XM_003988067.6
	R: ATCAAGCGCCTGGATTTGGA		
*GAPDH*	F: TGTGAACGGATTTGGCCGTA	174	NM_001009307.1
	R: CCGTTCTCAGCCTTGACTGT		

IL-8 = Interleukin-8, TLR8 = Toll-Like Receptor 8, TLR4 = Toll-Like Receptor 4, MyD88 = Myeloid Diff erentiation Primary Response 88, KLF6 = Kruppel-Like Factor 6, GAPDH = Glyceraldehyde 3-phosphate dehydrogenase

### Statistical analysis

To confirm statistical significance, the *p*-value and z-score were computed using the computational algorithms of the Student’s t-test and Fisher’s exact test. Statistical analyses for RT-qPCR data were performed using Prism 8 software(GraphPad Software, San Diego, CA, USA). Data were represented as mean ± Standard Deviation (SD), and all experiments were conducted in three independent replicates, with each performed in triplicate.

## Results

### Differential mRNA expression in the PBMCs between normal, FIPD, and FIPR groups

**Fig. 1:**
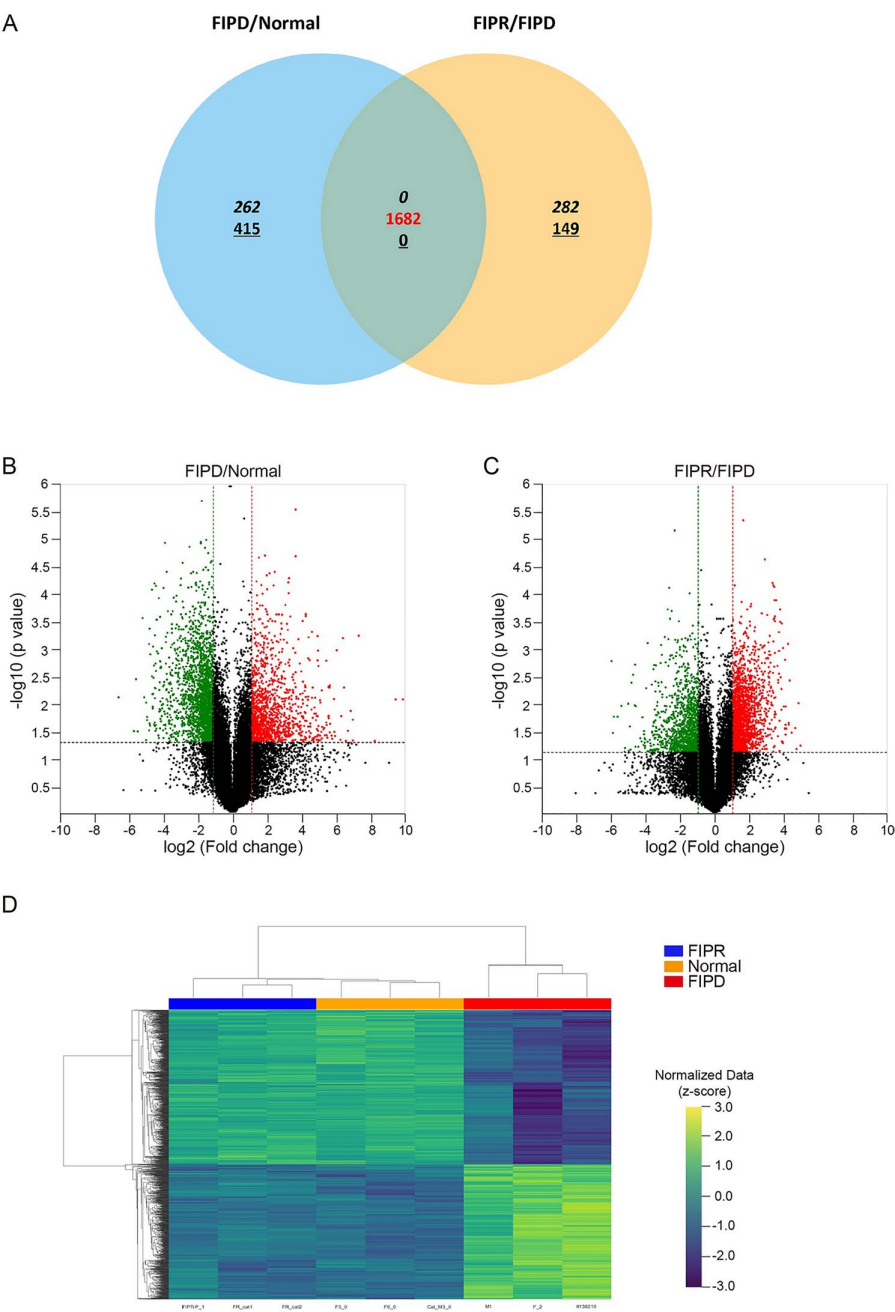
Summarized Transcriptome profiling of PBMCs between FIPD/Normal and FIPD/FIPR comparisons (**A**) Venn diagram shows the common up- (Italic), down- (Dashed), and contrast-regulated (Red) genes between FIPR/FIPD and FIP/Normal comparisons. Volcano plots representing DEGs pattern between (**B**) FIPD/Normal and (**C**) FIPR/FIPD. X-axis indicates log_2_ FC, and Y-axis indicates -log (*p*-value). Up (red) and down (green) regulated genes were filtered based on statistical significance (*p* < 0.05,|log_2_ fold| ≥ 1). (**D**) Clustering heatmap showing the gene expression profiles of individuals in FIPR, Normal, and FIPD groups

To examine unbiased gene expression in the PBMCs of Normal, FIPD, and FIPR cats, mRNA sequencing was conducted. A total of 16,733 genes were detected from the 31,503 genes in the feline reference genome. In the FIPD/Normal comparison, a total of 677 DEGs (262 upregulated and 415 downregulated) were identified with statistical significance (*p* < 0.05,|log_2_ FC| ≥ 1). In the FIPR/FIPD comparison, 431 DEGs (282 upregulated and 149 downregulated) were identified with statistical significance. Comparing these two comparisons, 1,682 DEGs were contrast-regulated (Fig. 1A). The volcano plot (Fig. 1B and C) depicted the distribution of DEGs in the FIPD/Normal and FIPR/FIPD comparisons, with statistical significance. A hierarchical clustering heatmap (Fig. 1D) revealed transcriptomic differences among the groups, and the individual sample variation in each group was assessed based on a principal component analysis (PCA) plot (Supplementary Fig. 1). Whereas Normal and FIPR groups were found to be characterized by similar gene expression profiles. We observed significantly different clusters for the FIPD Group. To elucidate changes in the biological function of PBMCs, we focused on the top 10% and bottom 10% DEGs between FIPD and Normal (Tables 2 and 3). Most of the DEGs in the Top 10% were related to immune-related pathways.

**Table 2:** Gene list of top 10% upregulated genes (FIPD/Normal)

Gene symbol	Log_2_ Fold change	Accession number	Gene description
G0S2	8.879	XM_011290970	G0 /G1 switch 2
PTGS2	6.583	NM_001110449	prostaglandin-endoperoxide synthase 2
PPP1R3B	6.164	XM_006930598	protein phosphatase 1 regulatory subunit 3B, transcript variant X1
PLAUR	6.009	XM_006941179	plasminogen activator, urokinase receptor, transcript variant X2
S100A9	5.903	XM_003999783	S100 calcium binding protein A9, transcript variant X2
PGLYRP1	5.756	XM_003997668	peptidoglycan recognition protein 1
TLR4	5.671	NM_001009223	toll like receptor 4
ALOX5	5.621	XM_003994181	arachidonate 5-lipoxygenase, transcript variant X1
NAMPT	5.432	XM_011280328	nicotinamide phosphoribosyltransferase
AATK	5.350	XM_023244281	apoptosis associated tyrosine kinase, transcript variant X3
WLS	5.316	XM_003990178	wntless Wnt ligand secretion mediator, transcript variant X1
AQP9	5.281	XM_003987104	aquaporin 9
PADI4	5.207	XM_003989527	peptidyl arginine deiminase 4, transcript variant X1
MARCKS	5.171	XM_023254273	myristoylated alanine rich protein kinase C substrate
ACSL3	5.105	XM_023259728	acyl-CoA synthetase long chain family member 3, transcript variant X2
BMX	5.083	XM_019823577	BMX non-receptor tyrosine kinase, transcript variant X4
LTF	5.074	XM_011291127	lactotransferrin
BCL2A1	5.019	XM_003986739	BCL2 related protein A1, transcript variant X3
CXCL8	4.997	NM_001009281	C-X-C motif chemokine ligand 8
ADGRG3	4.942	XM_019819797	adhesion G protein-coupled receptor G3, transcript variant X1
PROK2	4.918	XM_023249960	prokineticin 2, transcript variant X1
SOD2	4.905	XM_023254547	superoxide dismutase 2
GADD45A	4.877	XM_023257813	growth arrest and DNA damage inducible alpha, transcript variant X1
FGD4	4.819	XM_023256914	FYVE, RhoGEF and PH domain containing 4, transcript variant X3
CD300E	4.740	XM_023244065	CD300e molecule
B9D1	4.676	XM_003996329	B9 domain containing 1
IL18RAP	4.652	XM_019826970	interleukin 18 receptor accessory protein, transcript variant X2
LRRC75B	4.650	XM_023241543	leucine rich repeat containing 75B
OSM	4.579	XM_023241580	oncostatin M, transcript variant X2
RUBCNL	4.576	XM_023251553	RUN and cysteine rich domain containing beclin 1 interacting protein like, transcript variant X9
MSRB1	4.575	XM_003999032	methionine sulfoxide reductase B1
CLEC5A	4.487	XM_019825931	C-type lectin domain containing 5 A, transcript variant X2
CLEC12A	4.482	XM_003988405	C-type lectin domain family 12 member A, transcript variant X1
NLRP12	4.479	XM_023245577	NLR family pyrin domain containing 12
MARCH3	4.479	XM_003980726	membrane associated ring-CH-type finger 3
SGK1	4.465	XM_019831272	serum /glucocorticoid regulated kinase 1, transcript variant X1
EHF	4.457	XM_019812258	ETS homologous factor, transcript variant X1
HCK	4.456	XM_006929858	HCK proto-oncogene, Src family tyrosine kinase
IL1B	4.448	NM_001077414	interleukin 1 beta
DMRTC2	4.442	XM_019819463	DMRT like family C2, transcript variant X7
SOCS3	4.431	XM_023244150	suppressor of cytokine signaling 3
SLCO4C1	4.410	XM_019837491	solute carrier organic anion transporter family member 4C1, transcript variant X3
SDR16C5	4.401	XM_003999843	short chain dehydrogenase /reductase family 16 C member 5
TRPM2	4.370	XM_019839301	transient receptor potential cation channel subfamily M member 2, transcript variant X2
PADI2	4.365	XM_003989524	peptidyl arginine deiminase 2
KCNJ2	4.360	XM_023243720	potassium voltage-gated channel subfamily J member 2, transcript variant X3
APOBR	4.306	XM_023247080	apolipoprotein B receptor
MREG	4.282	XM_023259667	melanoregulin
IL27	4.247	XM_003998744	interleukin 27, transcript variant X2
ADGRE1	4.237	XM_023240916	adhesion G protein-coupled receptor E1
TRIB1	4.196	XM_004000118	tribbles pseudokinase 1
LITAF	4.192	XM_023246210	lipopolysaccharide induced TNF factor
PANK1	4.181	XM_023240710	pantothenate kinase 1, transcript variant X3
MMP27	4.151	XM_006936578	matrix metallopeptidase 27
ARHGEF37	4.150	XM_006927999	Rho guanine nucleotide exchange factor 37
GPAT3	4.149	XM_006930997	glycerol-3-phosphate acyltransferase 3, transcript variant X3
RAB3IL1	4.128	XM_006937396	RAB3A interacting protein like 1, transcript variant X2
PLEKHG3	4.078	XM_011283267	pleckstrin homology and RhoGEF domain containing G3, transcript variant X4
FPR2	4.063	XM_023245239	formyl peptide receptor 2, transcript variant X1
EGR1	4.062	XM_003980809	early growth response 1
UPP1	4.023	XM_019825232	uridine phosphorylase 1, transcript variant X3
PHACTR1	4.014	XM_019830229	phosphatase and actin regulator 1, transcript variant X3
ACVR1B	4.011	XM_023257090	activin A receptor type 1B, transcript variant X1
SRGN	3.968	XM_003993998	serglycin
GRAMD4	3.957	XM_023257634	GRAM domain containing 4
NFIL3	3.945	XM_023243253	nuclear factor, interleukin 3 regulated, transcript variant X2
ERG	3.881	XM_023238738	ERG, ETS transcription factor, transcript variant X3
IRAK3	3.866	XM_023257205	interleukin 1 receptor associated kinase 3, transcript variant X2
FLOT2	3.857	XM_011288993	flotillin 2, transcript variant X2
LTB4R	3.840	XM_019832870	leukotriene B4 receptor, transcript variant X3
HS3ST6	3.827	XM_019821200	heparan sulfate-glucosamine 3-sulfotransferase 6, transcript variant X3
FOS	3.827	NM_001009341	Fos proto-oncogene, AP-1 transcription factor subunit
STAC	3.822	XM_011286319	SH3 and cysteine rich domain, transcript variant X2
RAB32	3.804	XM_003986627	RAB32, member RAS oncogene family
NAPSA	3.799	XM_003997470	napsin A aspartic peptidase
NCF4	3.791	XM_003989234	neutrophil cytosolic factor 4
PLIN2	3.789	XM_023242345	perilipin 2, transcript variant X1
WDFY3	3.785	XM_023252965	WD repeat and FYVE domain containing 3, transcript variant X3
IL1RAP	3.768	XM_023260308	interleukin 1 receptor accessory protein
ICAM3	3.746	XM_023245246	intercellular adhesion molecule 3
KLHL25	3.729	XM_023254940	kelch like family member 25, transcript variant X3
PEAK3	3.723	XM_023243395	PEAK family member 3, transcript variant X3
IDO1	3.708	XM_003984814	indoleamine 2,3-dioxygenase 1
RHOB	3.681	XM_003984470	ras homolog family member B
PLD1	3.677	XM_023260401	phospholipase D1, transcript variant X4
SLC15A3	3.667	XM_023239889	solute carrier family 15 member 3, transcript variant X1
IL36G	3.659	XM_019827573	interleukin 36 gamma, transcript variant X2
KCND2	3.654	XM_011280521	potassium voltage-gated channel subfamily D member 2
TMEM132A	3.652	XM_023239887	transmembrane protein 132 A, transcript variant X1
AGTRAP	3.635	XM_006934279	angiotensin II receptor associated protein, transcript variant X1
METRNL	3.620	XM_023244384	meteorin like, glial cell differentiation regulator
RPS6KA2	3.614	XM_023254569	ribosomal protein S6 kinase A2, transcript variant X2
GNG10	3.600	XM_023242567	G protein subunit gamma 10
LIMK2	3.598	XM_003994812	LIM domain kinase 2, transcript variant X1
PFKFB4	3.589	XM_003982163	6-phosphofructo-2-kinase /fructose-2,6-biphosphatase 4, transcript variant X2
SPI1	3.577	XM_019812399	Spi-1 proto-oncogene, transcript variant X2
TNFRSF1A	3.574	NM_001009361	TNF receptor superfamily member 1 A
ULK1	3.537	XM_023241256	unc-51 like autophagy activating kinase 1
ITGB2	3.530	XM_011285804	integrin subunit beta 2
FGR	3.528	XM_011284511	FGR proto-oncogene, Src family tyrosine kinase, transcript variant X4
MRAS	3.514	XM_023260531	muscle RAS oncogene homolog, transcript variant X1
PHLDA1	3.502	XM_004001594	pleckstrin homology like domain family A member 1

^1^Selection criteria for DEG is more than 2 fold change of gene expression and statistical significance threshold *p < 0.05

**Table 3:** Gene list of top 10% downregulated genes (FIP/Normal)

Gene symbol	Log_2_ Fold change	Accession number	Gene description
ALDOC	-5.914	XM_003996488	aldolase, fructose-bisphosphate C
LGALSL	-5.111	XM_023251713	galectin like
MPL	-5.009	XM_019836840	MPL proto-oncogene, thrombopoietin receptor, transcript variant X4
AVIL	-4.917	XM_011284080	advillin, transcript variant X2
TMEM47	-4.671	XM_011291679	transmembrane protein 47
MPIG6B	-4.572	XM_019830370	megakaryocyte and platelet inhibitory receptor G6b, transcript variant X4
GP9	-4.467	XM_019825143	glycoprotein IX platelet, transcript variant X3
TSFM	-4.436	XM_023257173	Ts translation elongation factor, mitochondrial, transcript variant X3
MYL9	-4.423	XM_003983556	myosin light chain 9, transcript variant X2
DNASE1	-4.331	XM_019821040	deoxyribonuclease 1
DUSP26	-4.330	XM_003984789	dual specificity phosphatase 26, transcript variant X1
ITGA2B	-4.300	XM_003996987	integrin subunit alpha 2b
MTURN	-4.294	XM_023250465	maturin, neural progenitor differentiation regulator homolog
GAS2L1	-4.289	XM_023241601	growth arrest specific 2 like 1
KIF2A	-4.260	XM_023256931	kinesin family member 2 A, transcript variant X1
CAPN5	-4.177	XM_023239405	calpain 5, transcript variant X1
PLEK2	-4.163	XM_003987746	pleckstrin 2
PDLIM1	-4.144	XM_023240760	PDZ and LIM domain 1
ADGRG1	-4.140	XM_019819790	adhesion G protein-coupled receptor G1, transcript variant X2
DIMT1	-4.137	XM_019834834	DIM1 dimethyladenosine transferase 1 homolog, transcript variant X3
CLIC5	-4.101	XM_011282370	chloride intracellular channel 5
MUC13	-4.099	XM_019839884	mucin 13, cell surface associated
RBPMS2	-4.085	XM_023255207	RNA binding protein with multiple splicing 2, transcript variant X2
EHD3	-4.068	XM_003984350	EH domain containing 3
MPC1	-4.062	XM_023254568	mitochondrial pyruvate carrier 1, transcript variant X2
TRAP1	-4.045	XM_023246827	TNF receptor associated protein 1, transcript variant X2
ITGB3	-4.032	XM_003997035	integrin subunit beta 3, transcript variant X2
MUSTN1	-4.032	XM_006928855	musculoskeletal, embryonic nuclear protein 1
LY6G6F	-4.031	XM_006931567	lymphocyte antigen 6 family member G6F, transcript variant X1
VWF	-4.020	NM_001246279	von Willebrand factor
NRGN	-3.988	XM_003992528	neurogranin
FAM210B	-3.978	XM_023251081	family with sequence similarity 210 member B, transcript variant X1
TREML1	-3.968	XM_006931693	triggering receptor expressed on myeloid cells like 1, transcript variant X1
CAVIN2	-3.965	XM_003990983	caveolae associated protein 2
ENDOD1	-3.964	XM_023239323	endonuclease domain containing 1
FBLN2	-3.950	XM_011280224	fibulin 2, transcript variant X2
FAM213A	-3.932	XM_023240612	family with sequence similarity 213 member A, transcript variant X2
GPX1	-3.927	XM_004001361	glutathione peroxidase 1
CC1H2orf88	-3.913	XM_019838376	chromosome C1 C2orf88 homolog, transcript variant X3
SPRN	-3.848	XM_023241171	shadow of prion protein, transcript variant X1
SEC14L5	-3.833	XM_003998895	Section 14 like lipid binding 5
NEXN	-3.816	XM_023258841	nexilin F-actin binding protein, transcript variant X2
MXRA7	-3.804	XM_023244060	matrix remodeling associated 7, transcript variant X1
MARCH2	-3.792	XM_023244497	membrane associated ring-CH-type finger 2, transcript variant X1
SMOX	-3.781	XM_023251425	spermine oxidase, transcript variant X5
TMEM40	-3.766	XM_019825103	transmembrane protein 40, transcript variant X6
EGFL7	-3.741	XM_019816758	EGF like domain multiple 7, transcript variant X2
MFAP3L	-3.741	XM_019828718	microfibril associated protein 3 like, transcript variant X3
ITGA2	-3.733	XM_023260785	integrin subunit alpha 2
IGFBP7	-3.721	XM_003985355	insulin like growth factor binding protein 7
REEP2	-3.717	XM_023255612	receptor accessory protein 2, transcript variant X1
CLEC1B	-3.715	XM_003988398	C-type lectin domain family 1 member B
YBX3	-3.705	XM_023256777	Y-box binding protein 3, transcript variant X1
EBF1	-3.698	XM_023260105	early B-cell factor 1
GP1BA	-3.665	XM_023243425	glycoprotein Ib platelet alpha subunit
GNG11	-3.659	XM_003982762	G protein subunit gamma 11
TSPAN9	-3.641	XM_023256682	tetraspanin 9, transcript variant X3
YPEL2	-3.615	XM_023244589	yippee like 2, transcript variant X1
DSTN	-3.610	XM_003983801	destrin, actin depolymerizing factor
SLC6A4	-3.606	XM_019817397	solute carrier family 6 member 4, transcript variant X2
XK	-3.604	XM_023249534	X-linked Kx blood group
ANGPTL1	-3.584	XM_003999231	angiopoietin like 1
CD79B	-3.574	XM_003997062	CD79b molecule, transcript variant X1
GUCY1A1	-3.567	XM_011281606	guanylate cyclase 1 soluble subunit alpha 1, transcript variant X2
RGS17	-3.540	XM_003986661	regulator of G protein signaling 17, transcript variant X1
SELENON	-3.532	XM_023258321	selenoprotein N
SSX2IP	-3.493	XM_023258862	SSX family member 2 interacting protein, transcript variant X4
RALGPS2	-3.490	XM_023247646	Ral GEF with PH domain and SH3 binding motif 2, transcript variant X2
WNK4	-3.477	XM_006940374	WNK lysine deficient protein kinase 4, transcript variant X3
PLEKHO1	-3.472	XM_023259107	pleckstrin homology domain containing O1, transcript variant X4
GJA4	-3.471	XM_003989842	gap junction protein alpha 4
IGSF3	-3.446	XM_023259039	immunoglobulin superfamily member 3, transcript variant X5
CD2H1orf198	-3.432	XM_023240426	chromosome D2 C1orf198 homolog
IDS	-3.415	XM_006944057	iduronate 2-sulfatase, transcript variant X3
PAX5	-3.413	XM_011288547	paired box 5, transcript variant X3
RGS10	-3.410	XM_023240996	regulator of G protein signaling 10, transcript variant X1
QRSL1	-3.386	XM_003986421	glutaminyl-tRNA synthase (glutamine-hydrolyzing)-like 1, transcript variant X1
BCR	-3.376	XM_003994832	BCR, RhoGEF and GTPase activating protein, transcript variant X1
PDGFD	-3.375	XM_023239055	platelet derived growth factor D, transcript variant X2
ITGA6	-3.365	XM_023259459	integrin subunit alpha 6
PDE5A	-3.340	XM_019828142	phosphodiesterase 5 A, transcript variant X3
DOK2	-3.311	XM_003984731	docking protein 2, transcript variant X1
PPP1R14A	-3.305	XM_023245504	protein phosphatase 1 regulatory inhibitor subunit 14 A
ESAM	-3.305	XM_003992530	endothelial cell adhesion molecule
ATP5F1E	-3.296	XM_023251059	ATP synthase F1 subunit epsilon, transcript variant X2
EXOC3L4	-3.282	XM_023256087	exocyst complex component 3 like 4, transcript variant X3
PAM	-3.276	XM_023258826	peptidylglycine alpha-amidating monooxygenase, transcript variant X13
EDAR	-3.275	XM_019826940	ectodysplasin A receptor, transcript variant X3
GP1BB	-3.271	XM_023242162	glycoprotein Ib platelet beta subunit
CMAH	-3.270	XM_011282051	cytidine monophospho-N-acetylneuraminic acid hydroxylase, transcript variant X3
TMEM189	-3.267	XM_023251114	transmembrane protein 189, transcript variant X3
CRYL1	-3.241	XM_003980288	crystallin lambda 1
PARVB	-3.234	XM_023257578	parvin beta, transcript variant X1
BACH2	-3.226	XM_019830960	BTB domain and CNC homolog 2, transcript variant X2
LHFPL2	-3.225	XM_023257794	LHFPL tetraspan subfamily member 2, transcript variant X3
DGKH	-3.215	XM_023251911	diacylglycerol kinase eta, transcript variant X4
FXYD4	-3.213	XM_023240696	FXYD domain containing ion transport regulator 4, transcript variant X2
GP5	-3.211	XM_023260294	glycoprotein V platelet, transcript variant X2
FAM98B	-3.210	XM_003987287	family with sequence similarity 98 member B
BCL11A	-3.204	XM_019827251	B-cell CLL /lymphoma 11 A, transcript variant X2
RASA3	-3.198	XM_023239448	RAS p21 protein activator 3
PARD6G	-3.194	XM_023242146	par-6 family cell polarity regulator gamma
KDR	-3.190	XM_011281879	kinase insert domain receptor
POLR2B	-3.185	XM_006931102	RNA polymerase II subunit B, transcript variant X2
XG	-3.174	XM_023249228	Xg blood group, transcript variant X9
CST3	-3.163	NM_001184972	cystatin C
ADGRA3	-3.149	XM_023253254	adhesion G protein-coupled receptor A3
SERPINB10	-3.146	XM_003995247	serpin family B member 10
TPD52	-3.141	XM_011291336	tumor protein D52, transcript variant X5
TUBB1	-3.135	XM_023250905	tubulin beta 1 class VI, transcript variant X1
LEF1	-3.134	XM_011281704	lymphoid enhancer binding factor 1, transcript variant X4
ABI2	-3.133	XM_019838510	abl interactor 2, transcript variant X12
FHL1	-3.127	XM_023249463	four and a half LIM domains 1, transcript variant X2
NBEA	-3.124	XM_023250717	neurobeachin, transcript variant X1
ARHGAP18	-3.117	XM_019831191	Rho GTPase activating protein 18, transcript variant X2
GUCY1B1	-3.113	XM_023252746	guanylate cyclase 1 soluble subunit beta 1
GPR174	-3.096	XM_019824022	G protein-coupled receptor 174
NCOA7	-3.092	XM_023254324	nuclear receptor coactivator 7
TIMP1	-3.090	XM_023249291	TIMP metallopeptidase inhibitor 1
STARD13	-3.084	XM_006927222	StAR related lipid transfer domain containing 13, transcript variant X4
SYN1	-3.082	XM_019823364	synapsin I, transcript variant X1
NRP2	-3.082	XM_006935486	neuropilin 2, transcript variant X1
PIK3C2B	-3.058	XM_023248052	phosphatidylinositol-4-phosphate 3-kinase catalytic subunit type 2 beta, transcript variant X2
STYK1	-3.057	XM_019834514	serine /threonine /tyrosine kinase 1, transcript variant X2
TIMP2	-3.017	XM_003997248	TIMP metallopeptidase inhibitor 2
SDK2	-3.013	XM_011289338	sidekick cell adhesion molecule 2
FGF1	-2.994	XM_011282709	fibroblast growth factor 1, transcript variant X7
TSPAN5	-2.993	XM_011281794	tetraspanin 5, transcript variant X1
GIN1	-2.978	XM_019837573	gypsy retrotransposon integrase 1, transcript variant X2
SELP	-2.976	XM_023247403	selectin P, transcript variant X9
TACC1	-2.967	XM_019828544	transforming acidic coiled-coil containing protein 1, transcript variant X1
KCNMA1	-2.960	XM_019813336	potassium calcium-activated channel subfamily M alpha 1, transcript variant X2
RASGRP1	-2.951	XM_023255509	RAS guanyl releasing protein 1, transcript variant X2
PDZK1IP1	-2.935	XM_019836948	PDZK1 interacting protein 1, transcript variant X2
BANK1	-2.934	XM_023252909	B-cell scaffold protein with ankyrin repeats 1
IL24	-2.930	XM_003999450	interleukin 24
KCNA3	-2.925	XM_019839096	potassium voltage-gated channel subfamily A member 3
FAM212B	-2.923	XM_023258981	family with sequence similarity 212 member B, transcript variant X2
TPM1	-2.919	XM_023255267	tropomyosin 1, transcript variant X10
TFPI	-2.914	XM_019838357	tissue factor pathway inhibitor, transcript variant X2
SMYD2	-2.909	XM_023248067	SET and MYND domain containing 2
EHD2	-2.893	XM_003997613	EH domain containing 2
AGPAT3	-2.890	XM_023238659	1-acylglycerol-3-phosphate O-acyltransferase 3, transcript variant X3
TOM1L2	-2.890	XM_006939908	target of myb1 like 2 membrane trafficking protein, transcript variant X1
ADARB1	-2.889	XM_023238631	adenosine deaminase, RNA specific B1, transcript variant X8
NREP	-2.888	XM_011284960	neuronal regeneration related protein
STARD4	-2.884	XM_006927876	StAR related lipid transfer domain containing 4, transcript variant X1
F2R	-2.871	XM_003981083	coagulation factor II thrombin receptor
NAP1L1	-2.867	XM_006933900	nucleosome assembly protein 1 like 1, transcript variant X5
SPP1	-2.864	XM_006930977	secreted phosphoprotein 1, transcript variant X2
ITGB1	-2.860	XM_023256252	integrin subunit beta 1, transcript variant X3
GRPR	-2.853	XM_019823588	gastrin releasing peptide receptor, transcript variant X2
AHNAK2	-2.853	XM_023256202	AHNAK nucleoprotein 2
FAM169B	-2.847	XM_023254965	family with sequence similarity 169 member B, transcript variant X2
SRD5A1	-2.842	XM_023239691	steroid 5 alpha-reductase 1
RND2	-2.838	XM_023243773	Rho family GTPase 2
F2RL3	-2.826	XM_023246905	F2R like thrombin or trypsin receptor 3, transcript variant X1

^1^Selection criteria for DEG is more than 2 fold change of gene expression and statistical significance threshold *p < 0.05

### Activated inflammatory signal in PBMCs of FIP cats

**Fig. 2:**
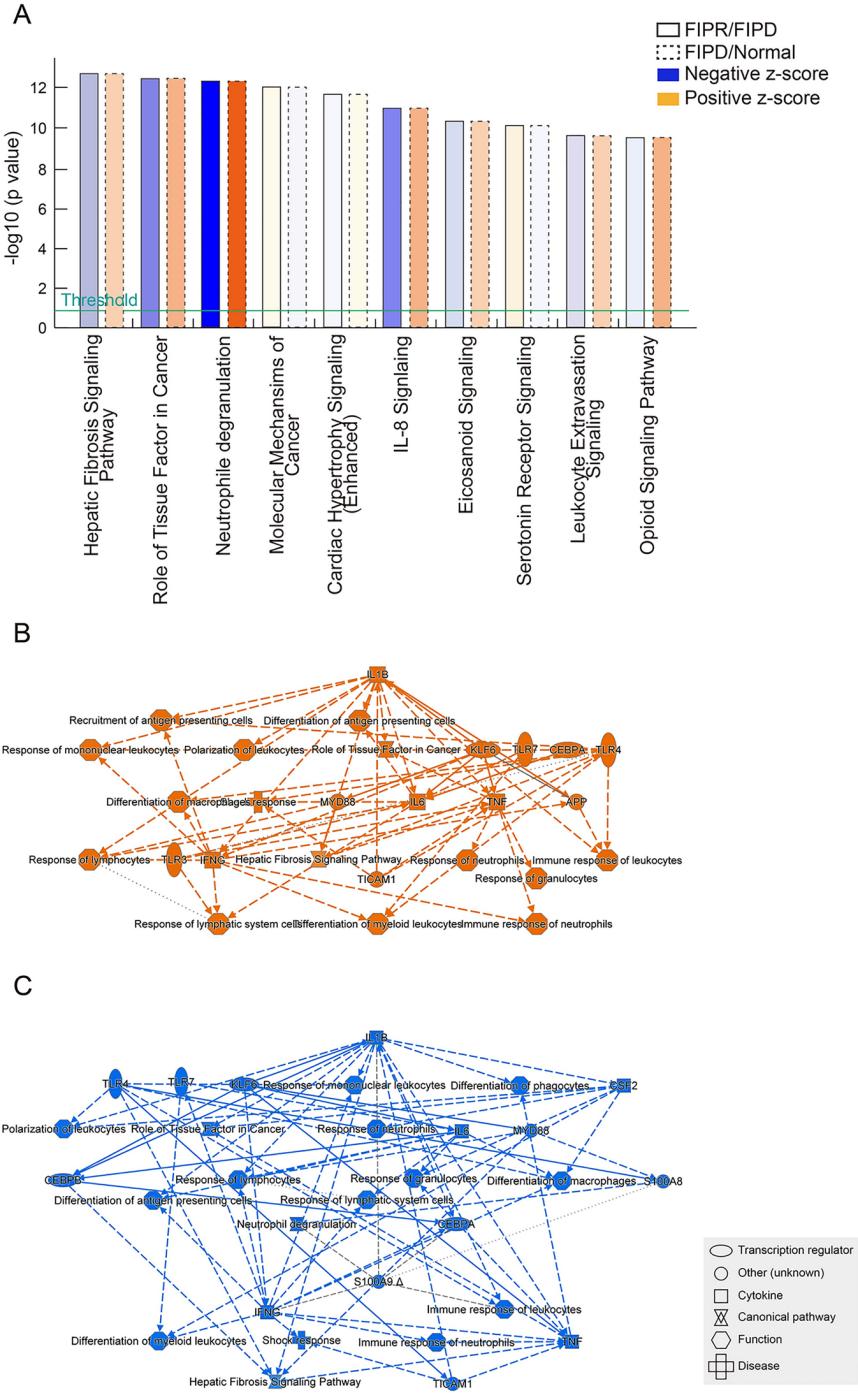
GO analysis from IPA software in PBMCs between FIPR/FIPD and FIPD/Normal comparisons (**A**) Top 10 canonical pathways ranked by -log (*p*-value). A negative z-score indicates an inhibitory signal in each pathway based on the DEGs. The significance threshold indicates -log (*p*-value) ≥ 1.3. Summarized molecular and pathway network of upregulated immune-related genes in PBMCs between (**B**) FIPD/Normal and (**C**) FIPR/FIPD. The color of the arrow and symbol indicates predicted activation (Orange), and inhibition (Blue)

**Fig. 3:**
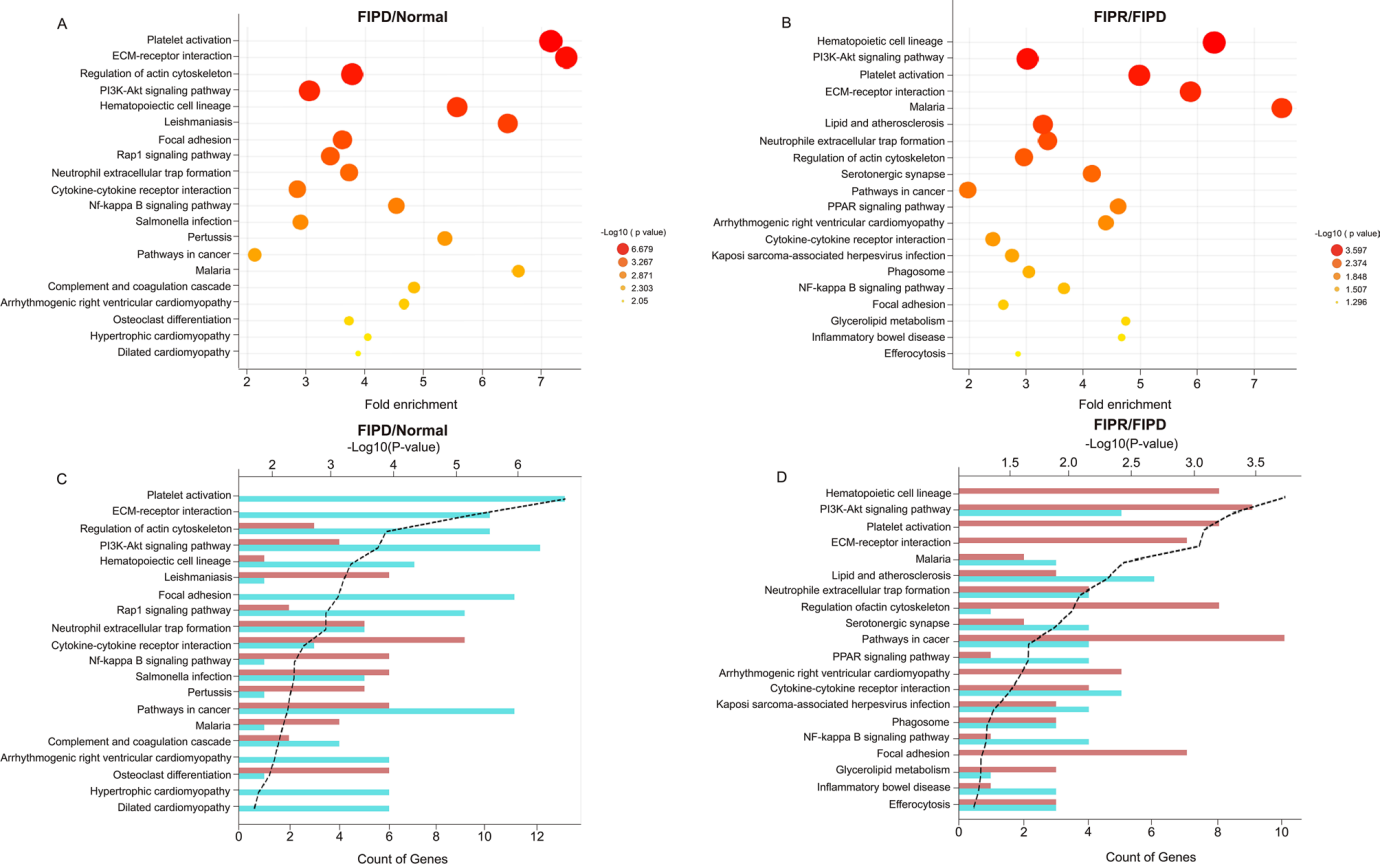
Top 20 of KEGG pathway enrichment analysis of DEGs ‘Fold enrichment’ and ‘Count of gene’ analysis in FIPD/Normal (**A**, **C**) and FIPR/FIPD (**B**, **D**) respectively. Functional annotation in the Y-axis is descending in order of -log (*p*-value) and the X-axis indicates fold enrichment and the count of up- and down-regulated genes associated with each pathway

Immune-related genes from PBMCs of the Normal, FIPD, and FIPR groups were analyzed using IPA and KEGG to identify the associated biological pathways. to identify the associated biological pathways, the top 10 biological pathways were filtered based on their high z-scores (Fig. 2A). Therefore, IPA was significantly enriched in various biological pathways. Among these, the neutrophil degranulation and IL-8 signaling pathways were identified as relevant and highly significant in the dataset. The neutrophil degranulation pathway demonstrated the highest -log (*p*-value) and z-scores, indicating statistical significance. A graphical summary of the IPA results highlighted the representation of inflammatory cytokines and their biological pathway**s** (Fig. 2B and C). Additionally, the IL-8 signaling pathway involved in neutrophil recruitment and degranulation was significantly enriched. The KEGG enrichment analysis indicated the top 20 enriched pathways for FIPD/Normal and FIPR/FIPD comparisons. Similar to the IPA analysis results, KEGG analysis indicated the enrichment of DEGs in the neutrophil-associated pathway, specifically in the neutrophil extracellular trap (NET) formation pathway (Fig. 3A and B). These enrichments were observed in both FIPD/Normal and FIPR/FIPD comparisons. However, the analysis of DEGs revealed no difference in the number of upregulated and downregulated genes (Fig. 3C and D).

### Activation of IL-8 by KLF6 and NF-κB signaling pathways

**Fig. 4:**
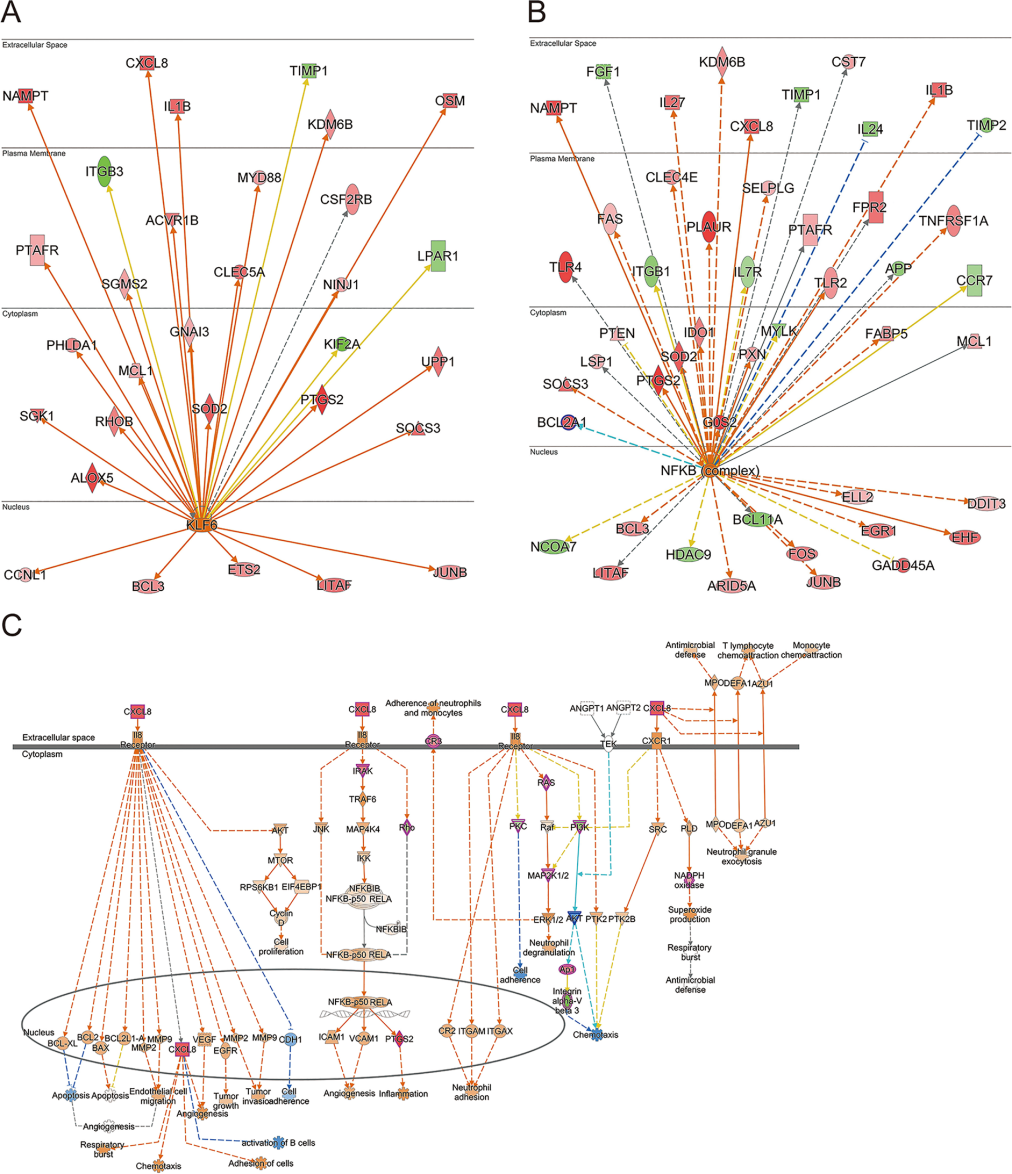
Activation of upstream molecules of IL-8 in FIPD/Normal comparison (**A**, **B**) Interaction of KLF-6 and NF-κB with downstream molecules in the cellular environment. In both analysis results, IL-8 was commonly detected as an activated molecule. (**C**) Comprehensive overview of IL-8’s role in FIP condition. The schematic representation demonstrates downstream events mediated by IL-8. The color of the symbol indicates predicted activation (Oragne), predicted inhibition (Blue), activation (Red), and inhibition (Green)

**Fig. 5:**
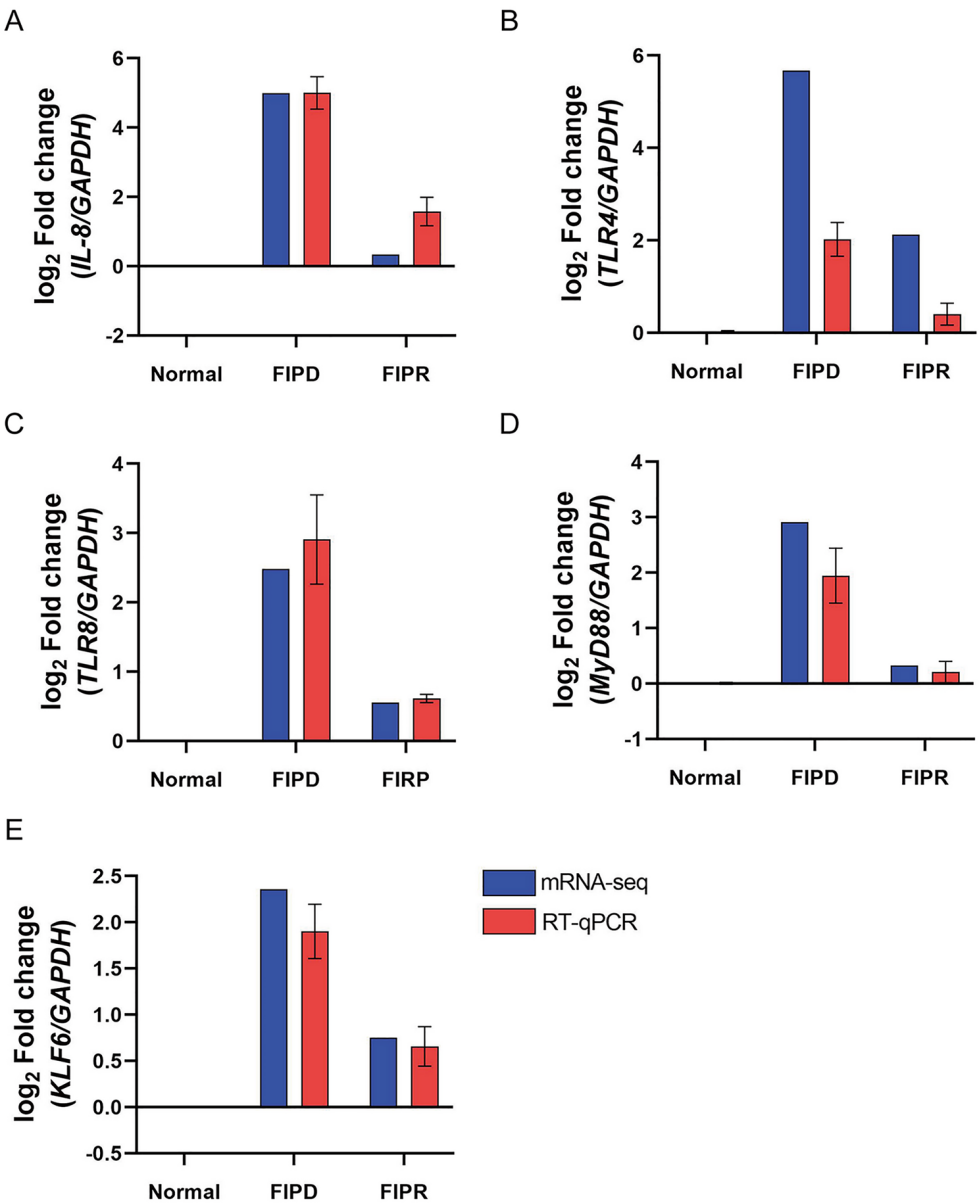
Validation of mRNA-sequencing data. Differential expression analysis of *IL-8*, *TLR4*, *TLR8*, *MyD88*, and *KLF6* across Normal, FIPD, and FIPR groups. Gene expression levels were quantified as log_2_ FC, comparing mRNA-seq (blue bars) and RT- qPCR (red bars) results. Panels display expression patterns for (**A**) *IL-8*, (**B**) *TLR4*, (**C**) *TLR8*, (**D**) *MyD88*, and (**E**) *KLF6*

Analysis of the upstream regulators and effectors of IL-8 revealed activation FIPD/Normal comparison. Specifically, KLF6 and NF-κB signaling pathways were identified as key upstream molecules of IL-8 activation (Fig. 4A and B). In the FIPD/Normal comparison, there was significant activation of the KLF6 and NF-κB signaling pathways (Fig. 4A and B), thereby upregulating IL-8 expression. Additionally, this activation was accompanied by upregulation of IL-8 and its associated downstream molecules (Fig. 4C). To confirm the mRNA sequencing data, RT-qPCR was conducted to assess the expression levels of *IL-8*,* Toll-like receptor 8 (TLR8)*,* Toll-like receptor 4 (TLR4)*,* myeloid differentiation primary response 88 (MyD88)*, and *KLF6* in Normal, FIPD, and FIPR groups. The expression levels were converted to log_2_ FC for comparison. The patterns of expression levels observed in the RT-qPCR results exhibited consistency with the mRNA sequencing data (Fig. 5).

## Discussion

FIP is an FCoV-caused disease, characterized by an immune-mediated response and high mortality rate, affecting both domestic and wild cats [15]. GS-441524 demonstrated high efficacy in cats with FIP by inhibiting viral replication and enhancing clinical symptoms [4, 16, 17]. However, there is a limited understanding of immune-related gene alterations during the progression and recovery of FIP. In this study, we conducted comparative transcriptome analyses of PBMCs obtained from Normal, FIPD, and FIPR cats to identify the underlying molecular mechanisms (Fig. 1). The analysis identified DEGs, indicating significant differences in gene expression patterns between Normal, FIPD, and FIPR cats (Fig. 1D). Based on these DEGs, we applied bioinformatics approaches to identify immune-related pathways that were altered during FIP progression and recovery.

The IPA identified the top 10 canonical pathways for both comparisons. Among these pathways, the neutrophil degranulation pathway was significantly activated in the FIPD/Normal comparison and inhibited in the FIPR/FIPD comparison. These findings indicate that neutrophils are activated during FIP progression and inhibited during recovery. Neutrophils are granule-filled white blood cells that play crucial roles during viral infection and inflammation, release granules to eliminate pathogens, and defend infection sites [18]. This process is mediated by various mediators, including cytokines such as IL-8 and TNF-α [19]. The IPA results notably demonstrated that the IL-8 signaling pathway was activated in the FIPD/Normal comparison and inhibited in the FIPR/FIPD comparison. These results are further supported by the mRNA sequencing data, which revealed an upregulation of IL-8 (log_2_ FC: 4.997) expression in the FIPD/Normal comparison, whereas IL-8 expression was downregulated in the FIPR/FIPD comparison. These findings thus confirm that the IL-8 signaling pathway acts as a chemoattractant and induces neutrophil migration, degranulation, and NET formation [20, 21].

The KEGG analysis further demonstrated significant enrichment in the NET formation pathway (Fig. 3). NET formation is a defense mechanism in which neutrophils release structures composed of DNA, histones, and antimicrobial proteins into their surroundings [22, 23]. NET formation can be triggered by various pathogens, including viruses [24–26]. This response neutralizes the invading pathogens, prevents their dissemination, and supports the immune response [27]. The NET formation process usually accompanies a specific form of programmed cell death referred to as NETosis [28]. NETosis induces the release of different pro-inflammatory mediators, including calgranulin proteins such as calgranulin A and B (S100A8 and S100A9), which contribute to the regulation of host inflammatory responses by interacting with pattern recognition receptors, such as the receptor for TLR4 [29, 30]. The mRNA sequencing data revealed a significant upregulation of S100A8 (log_2_ FC: 5.781), S100A9 (log_2_ FC: 5.903), and TLR4 (log_2_ FC: 5.671) in the FIPD/Normal comparison, thereby providing evidence for their roles in inducing inflammation and neutrophil activation.

To further dissect the regulartory mechanisms underlying IL-8 activation, we investigated the molecules upstream of IL-8 based on IPA. The IPA predicted that the upstream regulators KLF6 and NF-κB were activated in the FIPD/Normal comparison and inhibited in the FIPR/FIPD comparison (Fig. 4). Also, the mRNA sequencing data indicated significant upregulation of KLF6 (log_2_ FC: 2.357) expression in the FIPD/Normal comparison. KLF6 activates NF-κB and IL-8, which are induced by TNF-α and IL-1β [31]. Although TNF-α expression was not observed in the mRNA sequencing data, IL-1β (log_2_ FC: 4.448) expression was significantly upregulated. Therefore, we concluded that KLF6 plays a crucial role in regulating NF-κB and IL-8 activation. To confirm NF-κB activation, we analyzed its activation through the TLR-MyD88 signaling pathway, due to the limitations of mRNA sequencing data in assessing NF-κB activation [32]. Given that FIP is an ss-RNA virus, we observed the expression levels of TLR7 and TLR8, which recognize ss-RNA viruses [33]. TLR7 expression maintained a similar level in the FIPD/Normal comparison, whereas TLR8 (log_2_ FC: 2.485) expression was upregulated. After confirming TLR7/8 expression, we observed that MyD88 expression increased (log_2_ FC: 2.909), suggesting the activation of NF-κB.

To validate these findings, we confirmed the gene expression of IL-8 and its upstream regulators KLF6, TLR4, TLR8, and MyD88, which mediates the inflammatory response induced by calgranulin proteins. As a result, we observed that IL-8, KLF6, TLR8, and MyD88 expression levels were significantly higher in the FIPD group than in the Normal group, while these levels returned to normal values in the FIPR group, consistent with the mRNA sequencing results (Fig. 5 and Supplementary Fig. 2).

Our findings in this study provide novel insights into the immunological changes occurring in PBMCs during FIP infection and recovery. We identified distinct activation of IL-8 signaling and neutrophil degranulation pathways regulated by NF-κB and KLF6 in FIP infection condition, providing evidence that neutrophil-mediated inflammation plays a central role in disease progression and resolution. Given that GS-441524 effectively reduces viral load and alleviates clinical symptoms, we speculate that its therapeutic effects may mediated via the regulation of inflammatory pathways, as a consequence of viral suppression [34, 35]. However, in this study, the therapeutic and direct side effects of GS-441524 were not experimentally evaluated, warranting further investigation.

## Conclusions

The results of this study demonstrate that during FIP progression, IL-8 signaling plays a crucial role in neutrophil activation and degranulation, while these immune responses are suppressed by the antiviral effects of GS-441524 treatment. Specifically, the upstream regulators of IL-8, KLF6, and NF-κB were inhibited, resulting in reduced IL-8 activation and inhibition of the neutrophil degranulation pathway. These findings provide valuable insights into the molecular mechanisms underlying FIP and support the immunomodulatory effects of GS-441524 in regulating the immune response during FIP infection.

## Electronic supplementary material

Below is the link to the electronic supplementary material.

Supplementary Material 1

Supplementary Material 2

Supplementary Material 3

## Data Availability

All experiment data during this study are included in this paper and supporting files.

## Abbreviations

A/G: Albumin/Globulin
Calgranulin A: S100A8
Calgranulin B: S100A9
DAVID: Database for Annotation, Visualization, And Integrated Discovery
DEGs: Differentially Expressed Genes
FCoV: Feline Coronavirus
FECV: Feline Enteric Coronavirus
FIP: Feline Infectious Peritonitis
FIPD: FIP-Diseased
FIPR: FIP-Recovered
GAPDH: Glyceraldehyde 3-Phosphate Dehydrogenase
IL-1β: IL-1 beta
IL-8: Interleukin-8
IPA: Ingenuity Pathway Analysis
KEGG: Kyoto Encyclopedia of Genes and Genomes
KLF6: Kruppel-Like Factor 6
log_2_ fold change: log_2_ FC
NET: Neutrophil Extracellular Trap
NF-κB: Nuclear Factor kappa-light-chain-enhancer of activated B cells
Normal: Normal
NET: Neutrophil Extracellular Trap
MyD88: Myeloid Differentiation Primary Response 88
PBMCs: Peripheral Blood Mononuclear Cells
PCA: Principal Component Analysis
RT-qPCR: Reverse Transcription Quantitative Real-Time PCR
ss-RNA: single-stranded RNA
TLR4: Toll-Like Receptor 4
TLR7: Toll-Like Receptor 7
TLR8: Toll-Like Receptor 8
TNF-α: Tumor Necrosis Factor-Alpha
