# Supplementary figures and images for: Comparative transcriptome analysis of PBMCs in cats diagnosed with and recovered from FIPV
Source: Lab Anim Res. 2025 Jun 13;41:18. doi: 10.1186/s42826-025-00247-5 (PMC12164134; doi:10.1186/s42826-025-00247-5)

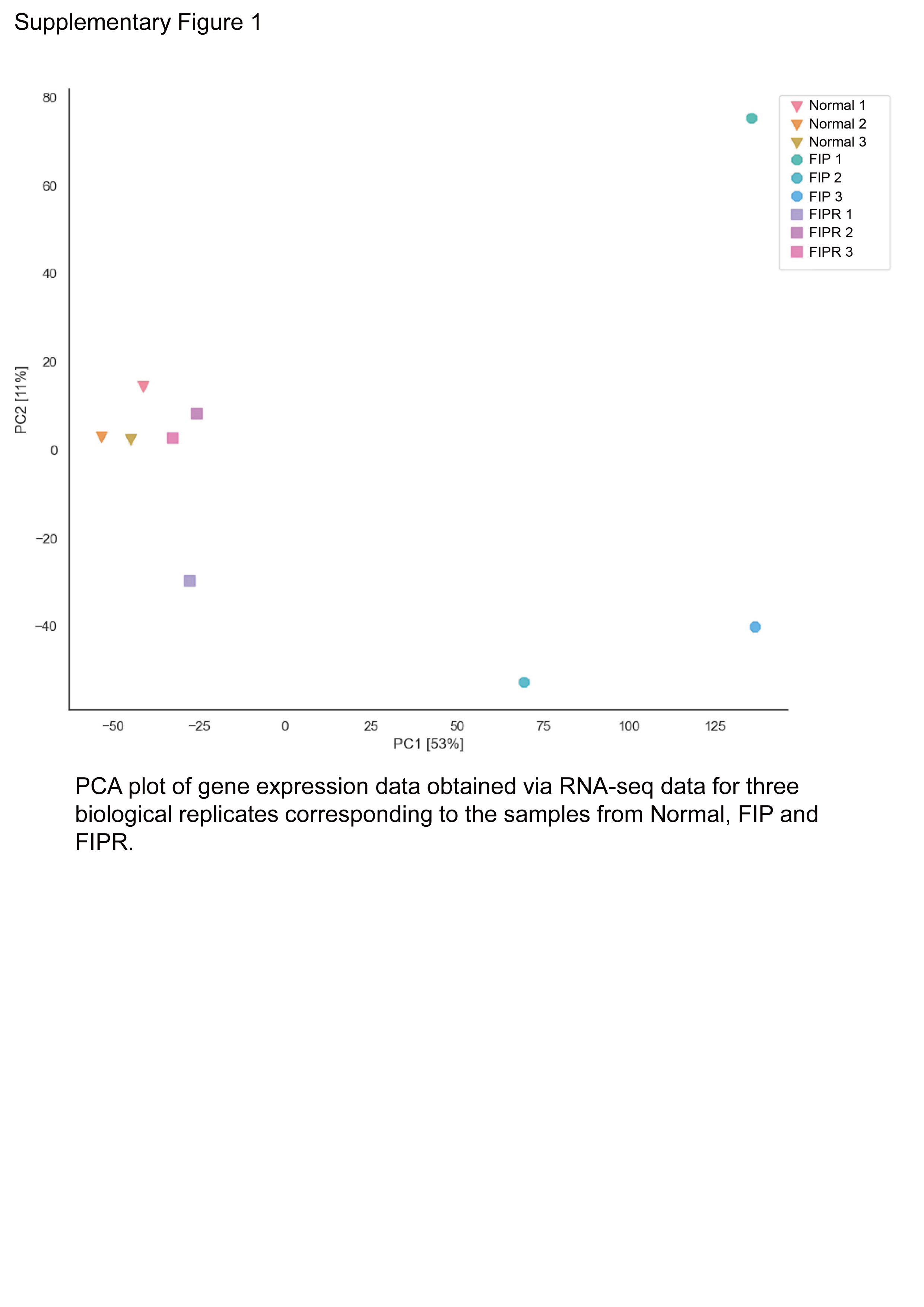

Supplement: Supplementary file 1 — Supplementary Material 1 [file 42826_2025_247_MOESM1_ESM.jpg]

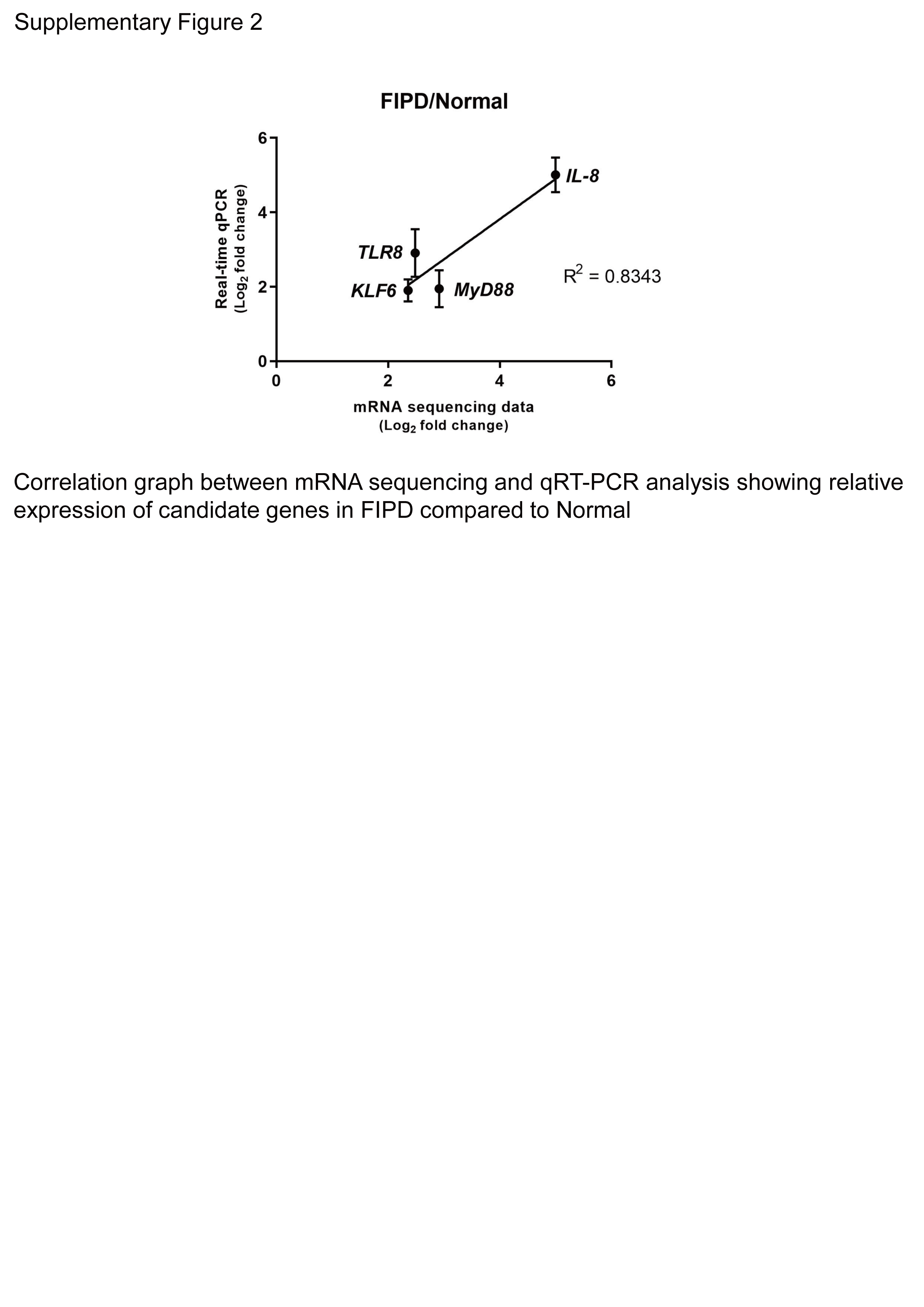

Supplement: Supplementary file 2 — Supplementary Material 2 [file 42826_2025_247_MOESM2_ESM.jpg]
