# Supplementary material for: Comparative transcriptome analysis of PBMCs in cats diagnosed with and recovered from FIPV
Source: Lab Anim Res. 2025 Jun 13;41:18. doi: 10.1186/s42826-025-00247-5 (PMC12164134; doi:10.1186/s42826-025-00247-5)
Supplement: Supplementary file 3 — Supplementary Material 3 [file 42826_2025_247_MOESM3_ESM.docx]

**Supplementary Table 1.** Information of Cats

| **Group** | **Breed** | **Age** | **A/G ratio**  **(< 0.4)** | **Rivalta test** | **Sex** |
| --- | --- | --- | --- | --- | --- |
| **Normal** | Korean Short hair | 7 months | X | Negative | Female |
|  | Korean Short hair | 6 years | X | Negative | Female |
|  | Korean Short hair | 5 years | X | Negative | male |
| **FIPD/FIPR** | Korean Short hair | 5 months | O | Positive | Female |
|  | Korean Short hair | 4 years | O | Positive | Female |
|  | Korean Short hair | 5 years | O | Positive | male |

A/G ratio: Albumin/globulin ratio
